# Exploring the Metabolic Stability of Engineered Hairy Roots after 16 Years Maintenance

**DOI:** 10.3389/fpls.2016.01486

**Published:** 2016-09-30

**Authors:** Suvi T. Häkkinen, Elisabeth Moyano, Rosa M. Cusidó, Kirsi-Marja Oksman-Caldentey

**Affiliations:** ^1^VTT Technical Research Centre of Finland Ltd.Espoo, Finland; ^2^Departament de Ciències Experimentals i de la Salut, Universitat Pompeu FabraBarcelona, Spain; ^3^Secció de Fisiologia Vegetal, Facultat de Farmàcia, Universitat de BarcelonaBarcelona, Spain

**Keywords:** plant cell culture, hairy roots, hyoscyamine 6β-hydroxylase, scopolamine, stability, cryopreservation

## Abstract

Plants remain a major source of new drugs, leads and fine chemicals. Cell cultures deriving from plants offer a fascinating tool to study plant metabolic pathways and offer large scale production systems for valuable compounds – commercial examples include compounds such as paclitaxel. The major constraint with undifferentiated cell cultures is that they are generally considered to be genetically unstable and cultured cells tend to produce low yields of secondary metabolites especially over time. Hairy roots, a tumor tissue caused by infection of *Agrobacterium rhizogenes* is a relevant alternative for plant secondary metabolite production for being fast growing, able to grow without phytohormones, and displaying higher stability than undifferentiated cells. Although genetic and metabolic stability has often been connected to transgenic hairy roots, there are only few reports on how a very long-term subculturing effects on the production capacity of hairy roots. In this study, hairy roots producing high tropane alkaloid levels were subjected to 16-year follow-up in relation to genetic and metabolic stability. Cryopreservation method for hairy roots of *Hyoscyamus muticus* was developed to replace laborious subculturing, and although the post-thaw recovery rates remained low, the expression of transgene remained unaltered in cryopreserved roots. It was shown that although displaying some fluctuation in the metabolite yields, even an exceedingly long-term subculturing was successfully applied without significant loss of metabolic activity.

## Introduction

### Plant Cell Cultures

Plants offer an enormous potential for humans in applications such as novel drugs, biopolymers, high-value chemical compounds, food and feed. Being ‘fully green,’ they possess the intrinsic capacity for environmentally friendly production processes and sustainability. Each plant cell has the whole genetic potential for differentiation into e.g., root, stem, leaf, or flower tissue. Besides whole plants, plant cells can be cultivated as cell cultures in synthetic growth media and this way they offer a great potential for various biotechnological applications. Plant cell cultures can be divided in two main classes, differentiated and undifferentiated cell cultures. The former consist of e.g., organs like shoots, roots or embryos, whereas callus and cell suspension cultures are referred to as undifferentiated cell cultures. Cell cultures offer advantages to produce cell biomass or metabolites in a controlled environment. Cultivation in laboratory or in bioreactors enables production processes which are independent of climatic conditions simultaneously allowing optimization of different production parameters.

Hairy root is caused by a plant disease through the infection of *Agrobacterium rhizogenes* carrying Ri (root inducing) plasmid. *Agrobacterium* (Rhizobiaceae) is a soil bacterium, which is able to deliver a part of its own plasmid-DNA (T-DNA) into the nuclear genome of the plant cell. As a result of very complex genetic machinery, hairy roots are then formed in the infection site. Finally they can be detached and grown as independent hairy root clones. Infection of a plant with *A. rhizogenes* is often associated with the induction of secondary metabolite biosynthesis (Sevón and Oksman-Caldentey, 2002). In the T-DNA there are four genetic loci, called *rolA*, *rolB*, *rolC*, and *rolD*, which are responsible for the hairy root phenotype and have been shown to positively affect the secondary metabolite production (Palazón et al., 1998; Bonhomme et al., 2000; Bulgakov et al., 2002). These genes can individually induce the secondary metabolite biosynthesis, with *rolB* probably being the most powerful inducer followed by *rolC* gene (Sharma et al., 2013). Although the exact mechanism of *rol* gene –mediated induction of secondary metabolite biosynthesis is not known, it is thought that induction involves the function of signal transduction pathways, including interaction with 14-3-3 proteins and tyrosine phosphatase (Moriuchi et al., 2004). In this work, *H. muticus* plant was infected with *A. rhizogenes* carrying hyoscyamine-6β-hydroxylase (*h6h*) from *H. niger*, a gene encoding for bifunctional enzyme responsible for tropane alkaloid hyoscyamine conversion into scopolamine (Hashimoto and Yamada, 1986). Hydroxylase activity of H6H leading to 6β-hydroxyhyoscyamine has commonly been observed to be much higher than the epoxidase activity leading to scopolamine (Yun et al., 1992).

Decades of research work has turned this hairy root disease into a valuable biotechnological application (Georgiev et al., 2007; Häkkinen et al., 2014; Vasilev et al., 2014). For a number of desired plants, hairy roots have been induced for the commercial scale production of metabolites, often yielding even higher amounts of metabolites than that of the parent plant (Jouhikainen et al., 1999; Häkkinen et al., 2005; Guillon et al., 2006; Srivastava and Srivastava, 2007). In addition, hairy roots gain biomass rather rapidly and have simple cultivation medium requirements.

### Stability of Hairy Root Cultures

Genetic stability of hairy roots is well recognized. They show high genetic stability as well as more stable metabolic production than that of undifferentiated cell cultures. In certain cases the production of secondary metabolites in plants is limited to specialized tissues or organs and the capability is lost in undifferentiated cells. This pattern is also recognized with tropane alkaloids, probably due to the root-specific localization of the tropane alkaloid biosynthetic pathway (Hashimoto and Yamada, 1986). Much of this stability has been related to chromosomic stability displayed by hairy roots (Weber et al., 2008, 2010; Dehghan et al., 2012). Hairy roots usually carry the same chromosomic number and karyotype as the parent plant. In addition, the fact that hairy roots are cultivated without additional plant growth regulators is likely to play a role, since when exposed to growth regulators, even organized tissues modify their chromosomic numbers and display somaclonal variation, as shown by Baíza et al. (1999). However, Guivarc’h et al. (1999) reported quite contradictory findings with hairy roots of *Daucus carota*, which were shown to be highly unstable both by phenotype as well as by their ability to express the transgenes during 2 years follow-up. Similarly, Mano et al. (1986) reported unstable production of tropane alkaloids in hairy roots of *Scopolia japonica*, although the follow-up was performed only for 2 months. However, this is a short period as the adaptation time to culture conditions is always required. Only few reports show the accumulation of metabolites produced by hairy roots during a long subculturing. Peebles et al. (2009) reported a 5-year study of hairy roots of *Catharanthus roseus* which were cultivated in laboratory and showed to be genetically and metabolically stable during this period. Similarly Maldonado-Mendoza et al. (1993) analyzed the tropane alkaloid production of hairy roots of *Datura stramonium* during 5 years and reported growth rates and alkaloid contents to be stable. In this work, we present, for the first time, the genetic and metabolic activities of engineered hairy roots maintained for 16 years by continuous subculturing. After the establishment of the hairy root line 16 years ago (Jouhikainen et al., 1999), it has been subjected to analysis of production capacity once in Häkkinen et al. (2005) before the current study.

### Cryopreservation

Biotechnology relies to a great part on working with cell cultures and continues to offer a future beyond depletion of natural resources. Establishment and maintenance of cell cultures is laborious and time-consuming process and so far only few successful methods exist for long-term storage for these cultures. Plant cells growing in undifferentiated state are known to be genetically unstable. During continuous subculturing, various rearrangements in chromosomal or gene level may take place, which can be of genetic or epigenetic origin (Larkin and Scowcroft, 1981; Kaeppler et al., 2000). Cryopreservation is a method where cells or tissues are stored in ultra-low temperatures, usually in liquid nitrogen (-196°C), halting all metabolic processes and thus allowing cells to retain their properties unchanged. However, cryopreservation of plant cells still mainly relies on the fully empirical testing, which makes the method development very laborious.

Basically, cryopreservation methods are divided in three categories, those based on slow freezing of the cells by using temperature gradients -0.3…-1.5°C/min, vitrification and encapsulation. The two latter ones do not require special freezing equipment but cells are immersed directly into liquid nitrogen after treatment with vitrification solutions or encapsulated with e.g., inside alginate in order to protect cell organelles from freezing injury. Optimization of intracellular water content is critical for cryopreservation success as plant cells may have water content up to 90%. Intracellular water expands during crystallization and may lead to bursting of cellular membranes. In addition, ice formation increases the solute concentration of remaining liquid phase in intracellular space which may cause toxicity during slow freezing. Prevention of intracellular water concentration may be overcome by several ways. Osmotic pretreatment means using non-permeable agents such as sugars and sugar alcohols which cause dehydration of cells. Cryoprotection on the other hand covers the use of permeable agents which function through a colligative effect and usually result in the increase in membrane fluidity. Freeze-induced dehydration is performed by controlled-rate cooling until terminal temperature -35…40°C is reached before samples are immersed into liquid nitrogen. Alternatively, when working with alginate bead protection, desiccation by air flow or silica gel is often used. When using vitrification-based techniques, dehydration is done by strong concentrations of osmotic agents and/or desiccation and cryoprotectives are included in vitrification solutions. However, vitrification solutions cause sometimes toxicity to plant cells and therefore are not suitable in all cases. Even though successful cryopreservation methods for a number of plant species have been established, a considerable experimentation is always needed to optimize all the steps in cryopreservation procedure (Reed, 2008). So far, there exists no routine method available for different species and cultures. Even though earlier reports of cryopreservation of hairy roots of *Beta vulgaris* and *Nicotiana rustica* (Benson and Hamill, 1991), *Artemisia annua* (Teoh et al., 1996), *Eruca sativa* and *Gentiana macrophylla* (Xue et al., 2008), *Panax ginseng* (Yoshimatsu et al., 1996), horseradish (Phunchindawan et al., 1997; Hirata et al., 1998), *Maesa lanceolata* and *Medicago truncatula* (Lambert et al., 2009) have been published, so far there exists no methods reported for cryostorage of *Hyoscyamus* hairy roots. Here, we present an easy method for cryopreservation of *H. muticus* hairy roots by using a simple vitrification procedure, allowing the freezing without complicated and expensive equipmentation. Furthermore, we present for the first time, the metabolite production of high scopolamine producing transformed hairy root clone of *Hyoscyamus muticus* after 16 years’ constant subculturing.

## Materials and Methods

### Plant Material

The hairy root clone KB7 (or formerly 13A7) of *H. muticus* (strain Cairo) was initiated by transforming with *A. rhizogenes* strain LBA9402 pLAL21 (carrying 35*S-h6h*) as described by Jouhikainen et al. (1999). The hairy roots were routinely grown and subcultured every 4 weeks in solid modified B50 medium (Oksman-Caldentey et al., 1991; Jouhikainen et al., 1999). In all the studies discussed in this paper, root inoculum was 100 ± 5 mg (FW) in 20 ml medium, and cultivation was performed in 100 ml erlenmeyer flasks, 100 rpm shaking, +25°C, 8 h/16h dark/light regime. Analysis for PCR, RT-PCR, and alkaloids were performed using 28 day-old roots cultivated in liquid culture medium. In order to verify the presence of the transgene as well as hairy root phenotype, the PCR analysis was performed for *h6h* (Häkkinen et al., 2005) and for *rolB* (Sevón et al., 1995). The absence of *virD* gene in hairy roots were verified using PCR primers described by Hamill et al. (1991) and amplification was performed as follows: initial denaturation at 95°C for 1 min, followed by 35 cycles of denaturation at 95°C for 30 s, annealing at 59°C for 1 min, and extension at 72°C for 1.5 min, followed by the final extension at 72°C for 7 min.

### RT-PCR

Hairy root samples for total RNA isolation were collected by immersing the roots in liquid nitrogen and storing the samples in -80°C before analysis. Total RNA from *H. muticus* hairy roots was isolated with the ARNzol kit (REAL, Valencia, Spain). cDNA was prepared by reverse-transcription with SuperScript II Reverse Transcriptase (Invitrogen, Carlsbad, CA, USA). The primer sequences for *18S* gene were 5′-ATGATAACTCGACGGATCGC-3′ and 5′-CTTGGATGTGGTAGCCGTTT-3′ (Zhang et al., 2007). The primer sequences for *h6h* gene were 5′- ACATCTGTGAAGGACTTGGGGC-3′ and 5′-GAACTTGGGTCTGGGCATGG-3′ designed using BT1 Gene Tool Lite (1.0.0.1 version). Real-time PCR was performed using the SYBR Green PCR Mastermix (Roche Applied Science, Mannheim, Germany) in a 384-well platform system (LightCycler 480 Instrument; Roche Applied Science). The *18S* gene was used as housekeeping control. Data were analyzed using the LightCycler480 Software release 1.5.0 SP3 (Roche). The statistical analysis was performed with the computing environment R (R Core Team, 2013). The student *T*-test was used for statistical comparisons. A *p*-value of less than 0.05 was set for significant differences.

### Tropane Alkaloid Extraction and Analysis

The tropane alkaloids scopolamine, hyoscyamine and 6β-hydroxyhyoscyamine were extracted from 28 days cultivated *H. muticus* hairy roots as described earlier (Häkkinen et al., 2005). Briefly, lyophilized roots were weighed (50 mg) and the lipids were removed with 2 ml petroleum ether. After vortexing and centrifugation (3000 rpm, 10 min) the solvent phase was discarded and the sample residue was dried under nitrogen flow. After adding H_2_O (2 ml) and 50 μg internal standard homatropine (Sigma), the samples were made alkaline (pH 9) and the alkaloids were extracted twice with 2 ml Cl_2_CH_2_. Before GC-MS analysis, the dried residue was dissolved in 100 μl Cl_2_CH_2_ and derivatized with MSTFA (*N*-methyl-*N*-trifluoroacetamide, Pierce). The tropane alkaloids from the culture medium were extracted correspondingly from 2 ml medium. Analyses were performed with an Agilent 7890A GC combined with a 5975C mass selective detector (MSD) using an Rtx^®^-5MS silica capillary column (15 m, 0.25 mm i.d., 0.25 μm phase thickness; Restek, Bellefonte, PA, USA). The oven temperature was increased from 70°C to 270°C at a rate of 10°C min^-1^. Helium was used as the carrier gas on constant flow mode at 1.2 mL min^-1^. The injector, ion source and interface temperatures were 250, 230, and 240°C, respectively, and the split ratio was 25:1. Samples (1 μL) were injected by a Gerstel Maestro MPS2 sampling system (Gerstel GmbH & Co. KG, Mülheim an der Ruhr, Germany). MSD was operated at electron-impact (EI) mode at 70 eV and the full scan data (m/z 40–600) was collected at a scan rate of 2.6 scans s^-1^. Identification of the compounds was based on retention times, GC-MS library comparisons and literature data. Chromatographic data were collected and evaluated using Chemstation Software (Agilent Technologies, USA).

### Cryopreservation

**FIGURE 1:**
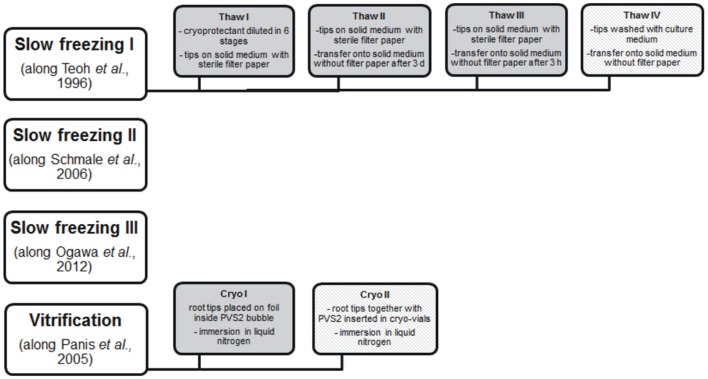
**Experimental layout of cryoprotection.** Deviations in original methods are described in section “Cryopreservation” and in gray-colored boxes. Methods which eventually showed post-thaw recovery are shown in patterned color.

Several cryopreservation methods were tested for long-term storage of *H. muticus* hairy roots including slow freezing and vitrification with different freezing profiles. Experimental layout is shown in **Figure 1.**

#### Slow Freezing I

Slow freezing method (Teoh et al., 1996) was tested for hairy roots of different growth stages (early exponential and stationary phase). Root tips were cut (0.5–1.0 mm) and incubated for 24 h on the solid medium. Cryoprotectant consisted of culture medium with 200 g/l sucrose and 8% (v/v) dimethylsulphoxide (DMSO). Root tips were transferred into cryo-vials and incubated at room temperature together with cryoprotectant for 1 h. Freezing program applied was: -10°C/min to + 4°C, -1.0°C/min to -35°C, hold 20 min. After that the vials were transferred into liquid nitrogen. Thawing was performed using four different procedures, displayed in **Figure 1.** First, the vials were thawed in +37°C water bath for 2 min and cryoprotectant was diluted in six stages in 10 min intervals fourfold with original culture medium. After incubation in culture medium for 1 h, root rips were placed on the solid medium with sterile filter paper and incubated in darkness. After 3 days the root tips were transferred on the solid medium without filter paper. In the second method, the vials were thawed in +37°C water bath for 2 min and root tips were placed on the solid medium and incubated as described earlier. The third method was as described above but root tips were transferred on solid medium without filter paper after 3 h incubation. Fourth, cryovials were thawed as described above; root tips were washed with original culture medium and plated on the solid medium without filter paper.

#### Slow Freezing II

Slow freezing method described by Schmale et al. (2006) was assayed with slight modifications. Hairy roots were cultivated in liquid medium for 7 days together with 50 μM abscisic acid. After precultivation, sucrose was added in a final concentration of 200 g/l and incubation was continued for 24 h. Root tips were cut and inserted into cryo-vials together with DGS solution (DMSO 1.6 M, glycerol 3.2 M, sucrose 0.4 M) at room temperature. Vials were incubated on ice for 20 min and frozen in CoolCell^®^ container before insertion in liquid nitrogen.

#### Slow Freezing III

Slow freezing procedure described by Ogawa et al. (2012) was assayed. Hairy roots were cultivated either on the solid medium for 4 weeks or in liquid medium for 6 days. After that the root tips were cut and cultivated on fresh solid plate for 24 h. Approximately 10 tips were placed on the cryo-vial together with 1 ml LSP solution (2 M glycerol, 0.4 M sucrose, 86.9 mM proline) and incubated for 1 h at room temperature. Cryo-vials were either placed on the Nalgene CoolCell^®^ container in -80°C, allowing the temperature gradient -1°C/min or frozen with slow freezing method: -5.0°C/min to 0°C, -0.3°C/min to -35°C, hold 20 min. After 4 h incubation, vials were placed in liquid nitrogen.

#### Vitrification

Hairy roots were cryopreserved along a modified method essentially described for banana apical meristems by Panis et al. (2005). Hairy roots were cultivated for 14 days on solid medium. Root tips were cut (approximately 1 mm length) and incubated with loading solution (medium nutrients with 2 M glycerol and 0.4 M sucrose) for 2 h. Loading solution was replaced by ice cold PVS2 solution (3.26 M glycerol, 2.42 M ethylene glycol, 1.9 M DMSO, 0.4 M sucrose with medium nutrients) and were incubated on ice for 30 min. Root tips were either placed on foil inside PVS2 bubble and were frozen directly in liquid nitrogen, or tips together with PVS2 were inserted in cryo-vials and immersed in liquid nitrogen.

#### Thawing

Root tips were thawed in a following way unless otherwise described. Cryovials were thawed in +37°C water bath for 2 min and root tips were placed on the solid medium with sterile filter paper. Tips were transferred on fresh solid medium without filter paper after 24 h and incubated in darkness.

## Results

### PCR and RT-PCR Showing the Genetic Stability of *h6h*-Transformed Root Cultures

**FIGURE 2:**
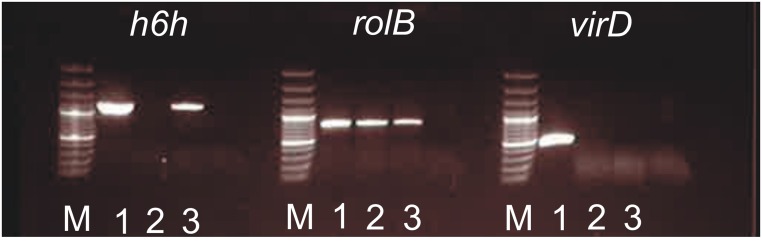
**PCR of *h6h* (1150 bp), *rolB* (780 bp), and *virD* (450 bp) amplifications performed of *Hyoscyamus muticus* hairy roots and *Agrobacterium* samples.** 1: *A. rhizogenes* pLAL21 carrying *h6h*; 2: *H. muticus* hairy root control; 3: *H. muticus* hairy root KB7 carrying *h6h*; M: Gene Ruler 100 bp Plus ladder.

PCR was performed using *h6h* gene-specific primers, and it was shown that both transgene and root phenotype responsible *rolB* region were present in *H. muticus* hairy roots after 16-year continuous subculturing (**Figure 2**). As expected, *virD* amplification, indicating the presence of the bacterial genome was negative.

**FIGURE 3:**
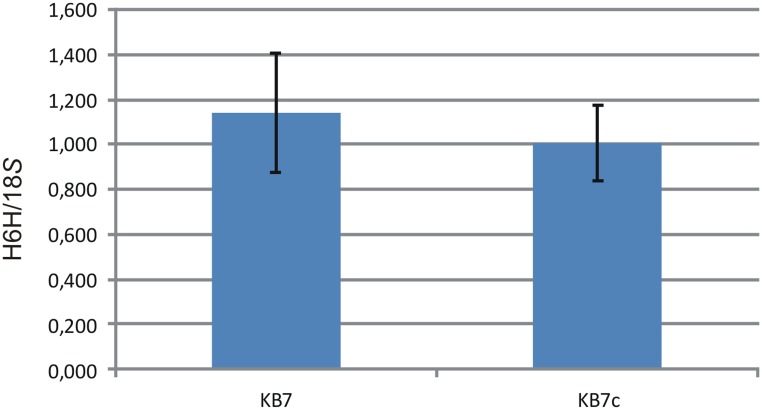
**Relative quantification results for the RT-PCR of H6H and 18*S* in the original (KB7) and cryopreserved (KB7c) samples.** Data are the mean of three analytical replicates (± SD).

RT-PCR of H6H expression was performed from original KB7 line and of that after cryopreservation (**Figure 3**). There was no significant difference in expression level of H6H in original and cryopreserved line (*p* = 0.8538).

### Growth and Stability of Metabolite Production

*Hyoscyamus muticus* KB7 hairy root clone was established in 1998 (Jouhikainen et al., 1999) and the clone has been cultivated since then in the laboratory by subculturing on fresh media every 4 weeks. As described 16 years ago, the hairy roots have preserved their light color and fast growth rate, multiplying the biomass 65–67 fold in 28 days.

**FIGURE 4:**
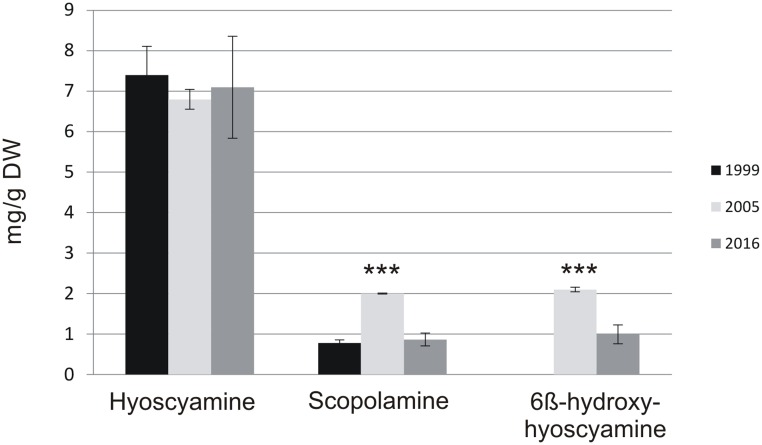
**Intracellular amount mg/g DW.** Asterisks indicate the statistical significance analyzed with Student’s *T*-test ^∗∗∗^*p* < 0.001. 6BHH: 6β-hydroxyhyoscyamine.

In **Table 1**, the accumulation of tropane alkaloids and growth during the 16-year follow-up is presented. First report of the KB7 line was published in Jouhikainen et al. (1999), with hyoscyamine and scopolamine production 125 mg/l and 14 mg/l, respectively. At that time, the hyoscyamine was determined by RIA (radio immuno assay) and scopolamine by ELISA (enzyme-linked immunosorbent assay), and the intermediate 6-β-hydroxyhyoscyamine was not measured. Six years later, we published a paper where conversion capacity of *h6h*-expressing *Nicotiana* and *Hyoscyamus* hairy roots were evaluated and in that study the endogenous production of the original KB7 was also measured. Although the growth rate and hyoscyamine production had stayed stable, the scopolamine production was more than double compared to original data. This result indicates that enzymatic activity of H6H at that time was for some unknown reason higher than before. Finally, last year the overall yields of alkaloids and biomass was somewhat lower than earlier (**Table 1**). Compared to the original data, hyoscyamine levels were 80% of the first reported values, whereas scopolamine content remained almost the same. However, when only cellular production capacity is taken into account (measured as mg/g DW) there are no differences between first and last measurement (**Figure 4**), and it is clear that the lower productivity shown in **Table 1** is a result of the lower biomass production, which reflects to the (mg/l) values.

**Table 1:** Accumulation of tropane alkaloids and growth of *H. muticus* KB7 hairy root clone during 16 years study period.

Year	Hyoscyamine (mg/l)	6β-hydroxy-hyoscyamine (mg/l)	Scopolamine (mg/l)	Growth (g/l DW)	Reference
1999^∗^	125 ± 12.0	NA	14 ± 1.3	17	Jouhikainen et al., 1999
2005^∗^	124 ± 6.2	38 ± 1.9	37 ± 1.9	17	Häkkinen et al., 2005
2016^∗∗^	99 ± 17.7	14 ± 3.3	12 ± 2.3	14	Current study


*Data represents biological replicates ± SD; ^∗^*N* = 3, ^∗∗^*N* = 9. NA: not analyzed.*

### Cryopreservation

**FIGURE 5:**
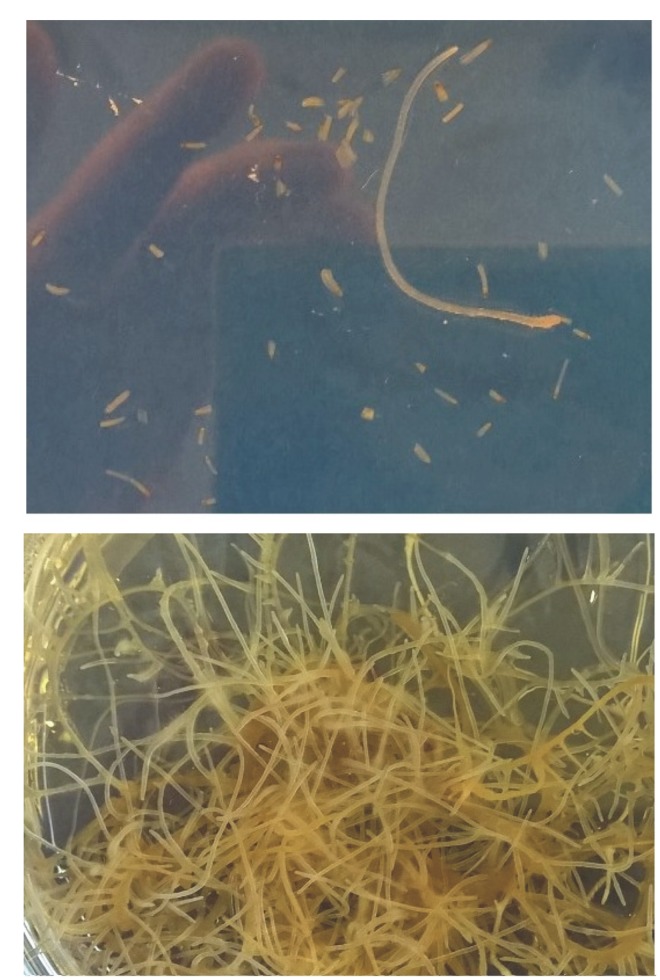
**Hairy roots cryopreserved with vitrification.** **(A)** 2 days, **(B)** 21 days after thawing.

Hairy roots cryopreserved with slow freezing I (section “Slow freezing I”) showed post-thaw recovery. Root tips thawed with the fourth method displayed post-thaw growth 2 weeks after thawing, however, with a relatively low recovery frequency (10%). Similarily, vitrification (section “Vitrification”) showed post-thaw recovery 2 days after thawing (**Figure 5**), but with even lower recovery frequency (∼2%). The recovered hairy root was then cultivated on a solid medium and H6H expression was analyzed after 21 days. As anticipated, cryopreserved root showed unaltered H6H expression levels as shown in **Figure 3** proofing the cryopreservation as a powerful tool for storing engineered biological material.

## Discussion

Often encountered problem with transgenic cell cultures is gene silencing, which is typically caused by transcriptional inactivation or post-transcriptional events (Guivarc’h et al., 1999). It is interesting to note that even if the *h6h* gene used in this study originates from the species of the same family as the host plant *Hyoscyamus*, gene silencing was not observed during the 16-year follow-up period. When the nucleotide sequences of five *Solanaceae* species possessing endogenous *h6h* genes were compared with *h6h* of *H. niger*, the similarity varied between the highest 99.2% (*Brugmansia candida*) to 84.9% (*D. stramonium*) (**Supplementary Table S1**). It is interesting to note that the highest similarity in protein level was observed with *B. candida* (100%), while that of another *Hyoscyamus* species *H. senescens* was somewhat lower, 96.5%. The sequence of *H. muticus h6h* is not known currently, but based on the sequence information of the related species; high similarity is expected as thus the possibility for homologous silencing event would be possible.

Being plant secondary metabolites, alkaloids are often produced in a low and unstable manner in undifferentiated plant cells. Secondary metabolite production has often been shown to correlate with the degree of organization (Sevón et al., 1998), thus hairy root tissue offers a promising platform for biotechnological alkaloid production which was also shown in the current study. For the first time the genetic stability coupled with metabolite production capacity of transgenic hairy roots was followed after 16 years continuous cultivation in laboratory. In this study, the alkaloid production of the one particular hairy root clone was measured in 1999, 2005, and 2016. The first analyses were performed using immunoassays while later the alkaloids were analyzed by GC-MS. While there is no experimental data presented about the correlation of the two analytical methods used, it should be noted that both EIA/RIA and GC-MS are highly specific and sensitive toward the metabolites analyzed, thus the data from the two analyses are comparable to justify the results.

Even though earlier reports of cryopreservation of hairy roots of certain plant species have been published, so far there exists no methods reported for cryostorage of hairy roots of *Hyoscyamus* sp. Recovery rates after cryostorage and thawing of hairy roots range from 6% (Xue et al., 2008) to 90% (Lambert et al., 2009), depending on the species and methods used. As Xue et al. (2008) reported, the method which was suitable for variable recovery for *E. sativa* and *Astragalus membranaceus* (73 and 6%, respectively), did not result in viable cells for *G. macrophylla* hairy roots. This phenomenon is commonly seen with plant cell and tissue culture cryopreservation resulting in step to step empirical testing for discovery of putative suitable cryostorage method.

Based on experiments described here, it is clear that cryopreservation of *H. muticus* hairy roots is very challenging and the results obtained so far are not sufficient for creating a reliable cryopreservation SOP. Method establishment for cryostorage of plant cells is of very empirical nature and often the results do not give clear directions for optimisation work. As stated by many researchers, the lack of reproducibility is a factor that limits routine application of cryopreservation (Reed et al., 2001; Panis et al., 2005). For organized tissues such as hairy root apical meristems, the labor intensive cutting and handling of small and fragile tissue parts is a clear obstacle for high-throughput testing. However, in this research, we could show that when successful, cryopreservation itself did not inhibit the transgene function in transgenic tissue. When applicable, cryopreservation is the best choice for maintaining plant cell and tissue cultures in unaltered state for basically unlimited time. This enables the much lowered risk of contamination and improves economic factors related to continuous cultivation. However, for organized tissues such as hairy roots shown in this study, long-term subculturing was successfully applied with genetically engineered roots without significant loss of enzymatic or metabolic activity.

## Author Contributions

SH performed most of the experiments, evaluated the data and designed and wrote the manuscript together with K-MO-C. EM performed the RT-PCR experiments, contributed to the writing. RC supervised the RT-PCR experiments, participated in planning the manuscript, and revised the manuscript. K-MO-C had an original idea of the stability and cryopreservation work, designed the work and wrote the manuscript together with SH.

## Conflict of Interest Statement

The authors declare that the research was conducted in the absence of any commercial or financial relationships that could be construed as a potential conflict of interest.
