# Supplementary material for: Exploring the Metabolic Stability of Engineered Hairy Roots after 16 Years Maintenance
Source: Front Plant Sci. 2016 Sep 30;7:1486. doi: 10.3389/fpls.2016.01486 (PMC5044514; doi:10.3389/fpls.2016.01486)
Supplement: Supplementary file 1 [file Table_1.DOCX]

**Table S1.** Sequence alignments of five *Solanaceae* *h6h*’s.
